# Did My Hand Move in a Mirror? Body Ownership Induced by the Mirror Hand Illusion

**DOI:** 10.3389/fnhum.2021.684873

**Published:** 2021-06-18

**Authors:** Akihiro Iida, Hidekazu Saito, Hisaaki Ota

**Affiliations:** ^1^Graduate School of Health Sciences, Sapporo Medical University, Sapporo, Japan; ^2^Department of Occupational Therapy, School of Health Sciences, Sapporo Medical University, Sapporo, Japan

**Keywords:** body ownership, mirror illusion, proprioceptive drift, persistence, questionnaire, healthy participant

## Abstract

Although the illusion that the mirror image of a hand or limb could be recognized as a part of one’s body behind the mirror, the effect of adding tactile stimulation to this illusion remains unknown. We, therefore, examined how the timing of tactile stimulation affects the induction of body ownership on the mirror image. Twenty-one healthy, right-handed participants (mean age = 23.0 ± 1.0 years, no medical history of neurological and/or psychiatric disorders) were enrolled and a crossover design was adopted in this study. Participants’ right and left hands were placed on the front and back sides of the mirror, respectively, then they were asked to keep looking at their right hand in the mirror. All participants experienced two experiments; one was with tactile stimulation that was synchronized with the movement of a mirror image (synchronous condition), and the other one was with tactile stimulation that was not synchronized (asynchronous condition). The qualitative degree of body ownership for the mirrored hand was evaluated by a questionnaire. Proprioceptive drift (PD), an illusory shift of the felt position of the real hand toward the mirrored hand was used for quantitative evaluation of body ownership and measured at “baseline,” “immediately after stimulation,” “2 min after stimulation,” and “4 min after stimulation.” The results of the questionnaire revealed that some items of body ownership rating were higher in the synchronous condition than in the asynchronous condition (*p* < 0.05). We found that PD occurred from immediately after to 4 min after stimulation in both conditions (*p* < 0.01) and there was no difference in the results between the conditions. From the dissociation of these results, we interpreted that body ownership could be elicited by different mechanisms depending on the task demand. Our results may contribute to the understanding of the multisensory integration mechanism of visual and tactile stimulation during mirror illusion induction.

## Introduction

In the context of self-consciousness, agency denotes the feeling of causing and controlling one’s action while body ownership refers to the sense of one’s own body—i.e., that one’s body belongs to oneself—which occurs with or without agency (Gallagher, 2000). The rubber hand illusion (RHI) paradigm is widely used to experimentally manipulate body ownership. During RHI, participants perceive a rubber hand placed near their real hand as their own when watching it being stroked synchronously with their unseen hand (referred to as the synchronous condition) (Botvinick and Cohen, 1998). In addition, the RHI can only be induced when the rubber hand movement is visually synchronized with that of the concealed real hand (Kalckert and Ehrsson, 2012; Riemer et al., 2013). Previous studies have found that concomitant tactile stimuli to the rubber and participants’ hands or synchronizing the movements of participants’ hand with those of the rubber hand are important for inducing RHI, and that if the stimulation or moving timing differs (referred to as the asynchronous condition), the RHI will either be mild or not occur at all (Botvinick and Cohen, 1998; Tsakiris and Haggard, 2005; Kalckert and Ehrsson, 2014; Shimada et al., 2014). The RHI causes participants to perceive the position of their real hand as closer to that of the rubber hand; this is called proprioceptive drift (PD) and can occur either with or without the participant’s awareness. PD is used as a quantitative indicator of the RHI effect (Tsakiris and Haggard, 2005). In addition, the RHI effect is qualitatively evaluated using a questionnaire (Botvinick and Cohen, 1998; Longo et al., 2008). However, whether a relationship between quantitative and qualitative results exists remains controversial (Longo et al., 2008; Holle et al., 2011; Rohde et al., 2011; Abdulkarim and Ehrsson, 2016).

The mirror illusion technique is another way to manipulate body ownership. It employs a mirror aligned with the midsagittal plane and participants are instructed to place one arm in front of the mirror and another one behind it. When participants’ observation is limited to the mirror image (visual exposure) or when they perform synchronized hand movements in front and behind the mirror while watching the mirror image (active movement), they perceive the mirror image as if it were the hand behind the mirror (Ramachandran et al., 1995; Holmes et al., 2004, 2006; Holmes and Spence, 2005). Previous studies used the bias in reaching movement by the concealed hand to evaluate the mirror illusion induction of body ownership. Medina et al. (2015) measured the perceived hand position similarly to RHI evaluations to assess the degree of mirror illusion. However, to the best of our knowledge, whether the mirror illusion technique can be induced using a passive tactile stimulation remains unknown. Furthermore, previous studies on body ownership documented the illusion occurrence but did not investigate its duration. Understanding these aspects may help elucidate the mechanisms involved in body ownership illusions.

Researchers involved in previous studies on the RHI used a rubber hand to investigate the induction of body ownership (Botvinick and Cohen, 1998; Tsakiris and Haggard, 2005; Longo et al., 2008; Holle et al., 2011; Rohde et al., 2011; Kalckert and Ehrsson, 2012, 2014; Riemer et al., 2013; Shimada et al., 2014; Abdulkarim and Ehrsson, 2016). Our methodology differed in that we induced an illusion on the image of a mirrored hand. Also, in a study that examined the induction of body ownership to the mirrored hand quantitatively, participants were required to actively move both their hands behind and in front of the mirror to facilitate induction (Medina et al., 2015). Our study differed in that we used passive tactile stimulation to facilitate induction. Furthermore, no studies have been performed to determine the persistence of the illusion thus far. In the RHI paradigm, it is vital that the tactile information from the participant’s hand matches the visual information garnered from the rubber hand being touched in order to induce the RHI (Botvinick and Cohen, 1998; Tsakiris and Haggard, 2005; Shimada et al., 2014). However, it has not been determined whether the illusion of body ownership could be transferred to the mirrored hand using tactile stimulation, and it is unclear how long the induced illusion lasts. When applying the RHI paradigm to the mirror illusion, we predicted that not only would the illusion be induced, but also that it would last for some time in the synchronous condition.

Therefore, this study aimed to clarify how synchronous or asynchronous tactile stimulation to the mirror image during mirror illusion technique affected body ownership illusion occurrence, and if the illusion occurred, whether it persisted. In addition, we also aimed to investigate the relationship between the induction of body ownership and its awareness. We hypothesized that (1) for inducing body ownership, the qualitative and quantitative evaluations would reveal that the synchronous condition is better than the asynchronous one, and that only the synchronous condition induces a lasting effect of body ownership; (2) a correlation between the qualitative and quantitative evaluation of body ownership would be found only in the synchronous condition.

## Materials and Methods

### Participants

Twenty-one right-handed healthy participants (12 female and nine male participants; mean age = 23.0 ± 1.0 years) participated in this study. The inclusion criteria were as follows; healthy volunteers, whose ages were from 20 to 35 and were right-handed, with a score of + 8 or higher in the Japanese version of the FLANDERS handedness questionnaire (Okubo et al., 2014). In addition, if any of the following seven items were applicable, they were excluded from participants; physical and/or cognitive dysfunctions that disturb task performance in our study, a poor vision which is difficult to correct and interferes with daily life, visual field defect, a current or medical history of neurological and/or psychiatric disorders, orthopedic disease or skin disease on both hands, experience with substance abuse or the use of hazardous substances and refusal to participate in the experiment or withdrawal of the consent.

### Sample Size

This experiment employed a 2 × 4 factorial design combining stimulation timing (synchronous and asynchronous) and measurement time (baseline, immediately after stimulation, 2 min after stimulation, and 4 min after stimulation). *A priori* power analysis was performed using G^∗^Power (Faul et al., 2007). The effect size was estimated to be moderate because there was not enough previous study (Cohen, 1988). We conducted *a priori* power analysis assuming a Cohen’s *f* = 0.25, α = 0.05, power = 0.80, Number of groups = 1, Number of measurements = 6, Correlation among repeated measures = 0.5, and Non-sphericity correction ε = 1. This yielded a total sample size of 19 and we enrolled 21 participants per condition as we adopted a crossover design and considered dropout.

### Procedure

#### Setting

**FIGURE 1:**
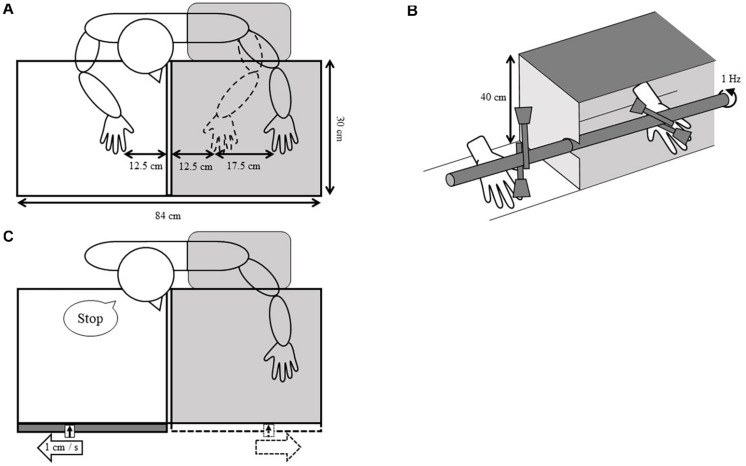
Experimental setup. **(A)** Hand position for the tactile stimulation. The participants sat on the chair, and their right hand was placed in the front of the reflective surface, whereas their left hand was positioned behind the mirror. The broken lines illustrate the mirror image seen by the participants. **(B)** Tactile stimulation device. Tactile stimulation was delivered by rotating a pole with the brushes. The angle of the brushes behind the mirror could be adjusted according to the stimulation conditions. This figure shows the asynchronous condition. **(C)** Finger point judgment task. The ruler with a marker was attached to the front of the mirror box. While looking at the mirror image of the horizontally moving marker, the participants verbally responded to the position where their left index finger and the marker vertically aligned. The broken lines illustrate the mirror image seen by the participants.

The experiment was conducted in a quiet room. The window curtain in the room was closed and common fluorescent lights on the ceiling were used to provide sufficient illumination to see their hand in the mirror throughout the experiment. The participants sat on a chair so that their midline was aligned with the center of the mirror box (W84 × D30 × H40 cm) that was placed on a table. A mirror (40 cm × 30 cm) facing to the right was positioned in the middle of the box. The participants’ hands were placed with palms facing down in the box so that their left and right index fingers were placed 30 cm behind the mirror and 12.5 cm in front of the mirror, respectively, and the positions of both index fingers are set parallel to the mirror (Figure 1A). The difference between the distance from the mirror to the left index finger and that from the mirror to the mirrored index finger was set at 17.5 cm because it was previously reported that the RHI effect is attenuated when the distance between the rubber hand and the participant’s hand is greater than 17.5 cm (Lloyd, 2007). The position of the chair was fixed after the participants adjusted it back and forth so that they could see the mirror image of their right hand and the range of movement of the marker for PD measurement well. The participants’ left upper limb was covered with a black curtain to prevent participants from seeing it. During the experiment, the participants were asked to relax and not move either of their hands until instructed by the experimenter.

#### Tactile Stimulation

Tactile stimulation was delivered to both index fingers from the metacarpal phalangeal joint to the fingertip using a homemade stimulation device with four identical brushes operated by the experimenter. The height and position of the stimulation device were adjusted for each participant to stroke the same part of their index fingers with the same intensity (Figure 1B). The experimenter heard a 1-Hz frequency sound from an earphone placed in one ear to allow them to operate the device at a 1-Hz stroke frequency. Based on a previous study, we employed a 2 min stimulation phase (Costantini and Haggard, 2007; Lane et al., 2017).

The participants experienced two conditions with different stimulation timings: the synchronous and asynchronous conditions. In the synchronous condition, the finger in the mirror and the participants’ left finger were stimulated at the same time; thus, the left and right index fingers were stroked simultaneously. Since previous studies showed that most participants detected a difference when the delay between visual and tactile stimuli was ≥500 ms (Shimada et al., 2014; Costantini et al., 2016), repeated tactile stimulation was given to one side approximately 500 ms following stimulus to the other side in the asynchronous condition as a control one. The participants were instructed to keep their gaze on the hand in the mirror and not to move their fingers during the stimulation period.

All participants performed both conditions, and the conditions were executed in a pseudo-random order to consider counterbalance. A 5-min rest period was set between trials to avoid the carryover effect.

#### Finger Position Judgment Task

We used a ruler and marker to measure PD as per previous studies (Tsakiris and Haggard, 2005; Shibuya et al., 2017; Smit et al., 2017). Each participant sat in front of the mirror box as described above, and the experimenter attached the ruler to the mirror box and sat in front of the participant across from the box (Figure 1C). The participants practiced the finger position judgment task as follows: They were asked to place their left hand behind the mirror and their right hand on their lap. Then, they were required to judge and report the perceived position of the visually occluded left index finger by projecting a parasagittal line from the mirror to the estimated fingertip position. They were required to respond verbally when they perceived that the sliding marker appeared to align with their index finger. The marker was manually moved at a speed of approximately 1 cm/s away from the mirror surface by the experimenter. The distance from the mirror surface to the aligned place was measured to the nearest mm. This practice was carried out five times in total, changing the position of the left hand arbitrarily.

After the practice, for the baseline measurement, the participants’ left index finger was placed 30 cm to the left, behind the mirror. The participants were instructed to keep this position and rest for 1 min to pay attention to the position of their index fingertip. This measurement was carried out once, as in the practice.

Immediately after the tactile stimulation, the experimenter removed the stimulation device and attached the ruler to the mirror box while asking participants to close their eyes and place their right hand on their lap, but not move their left hand. The experimenter asked the participants to open their eyes and measured the estimated position at which the marker aligned with the participants’ left index fingertip as the “immediately after stimulation” measurement. In addition, this measurement was repeated 2 and 4 min after stimulation in the same way. The measurement method was the same as in the baseline measurement and the participants closed their eyes between the measurements.

#### Questionnaire

After completion of all trials, the participants answered a questionnaire regarding body ownership for each condition. The questionnaire comprised four items (Table 1) based on previous studies (Botvinick and Cohen, 1998; Ide and Wada, 2016). The participants reported their subjective experience during each stimulation condition *via* a 7-point Likert scale, ranging from 1 (disagree strongly) to 7 (agree strongly).

**TABLE 1:** Questionnaire.

**Question**
1. It seemed as if I were feeling the touch of the paintbrush in the location where I saw the mirrored hand touched
2. It seemed as though the touch I felt was caused by the paintbrush touching the mirrored hand
3. I felt as if the mirrored hand were my left hand
4. I felt as if my hand were drifting toward the mirrored hand

*The questionnaire has been partially excerpted and reorganized based on cited references (Botvinick and Cohen, 1998; Ide and Wada, 2016).*

### Statistical Analysis

No dropouts were found in this study, and data from all participants were used for analysis. Questionnaire data for each item rating in each condition were analyzed by the Wilcoxon signed-rank test. The distance from the mirror to the estimated left index finger position (PD data) was analyzed by a two-way [stimulation condition (2) × measurement time (4)] repeated measures ANOVA. Greenhouse-Geisser correction was used where appropriate, and in such cases, we report the uncorrected degrees of freedom, the corrected *p* values, and the correction factor ε. The *post hoc* test was analyzed by Sidak’s multiple comparison test. Spearman rank correlation analysis was performed between the ratings of all questionnaire items and PD values to clarify the correlation between the qualitative and quantitative changes in body ownership. PD values were calculated by subtracting the positions of each of the three measurements after the stimulation from that of the baseline. Positive and negative values represent approaching and moving away from the mirror, respectively. Data were analyzed with SPSS Statistics version 25 (IBM Corp., Armonk, NY, United States) and *p* values < 0.05 were considered statistically significant.

## Results

### Questionnaire

**FIGURE 2:**
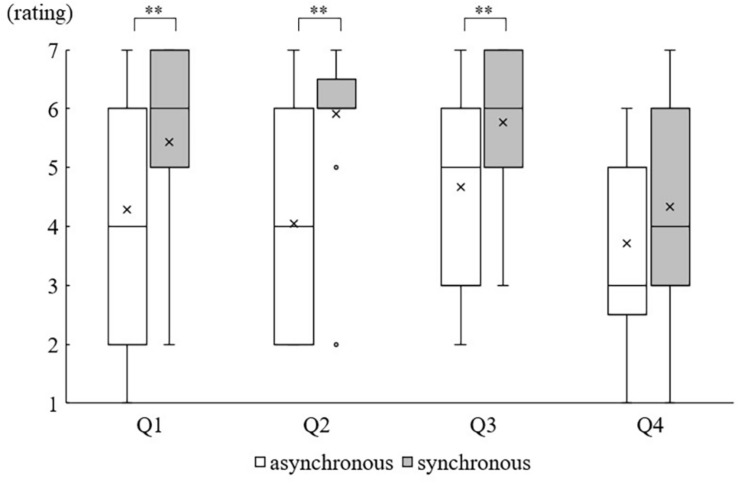
Questionnaire results: Boxes display interquartile data (IQR). Whiskers represent either extra data points or extend to 1.5 times IQR. Median and Mean are represented as horizontal lines and crosses within the box, respectively. The small circle denotes the outlier outside the whisker. ^∗∗^*p* < 0.01.

The Wilcoxon signed-rank test showed that the body ownership ratings of three items except Q4 were significantly higher in the synchronous condition than in the asynchronous condition (Q1, *Z* = −2.668, *p* = 0.008; Q2, *Z* = −3.443, *p* = 0.001; Q3, *Z* = −2.841, *p* = 0.004; Q4, *Z* = −1.912, *p* = 0.06) (Figure 2).

### PD

The normality of the distribution of the PD data from all measurement time points was assessed with the Shapiro–Wilk test (*p* ≥ 0.05). Mauchly’s test of sphericity showed a significant difference for measurement time (*p* < 0.0005) and stimulation condition × measurement time (*p* = 0.04).

**FIGURE 3:**
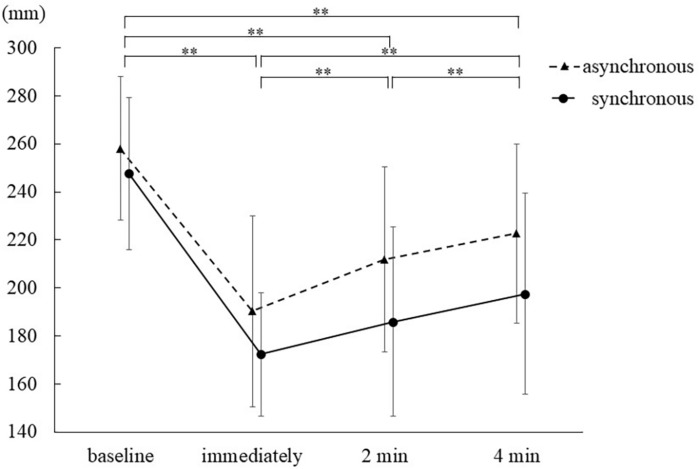
Proprioceptive drift results. ^∗∗^*p* < 0.01.

For PD data, a two-way repeated measures ANOVA demonstrated that the main effect of the measurement times was significant (*F*_1__.838__,36__.752_ = 92.527, *p* < 0.0005, η_*p*_^2^ = 0.822), but that of the stimulation condition was not (*F*_1,20_ = 2.841, *p* = 0.11, η_*p*_^2^ = 0.124). No interaction between these two factors was found (*F*_2__.260__,45__.197_ = 2.916, *p* = 0.06, η_*p*_^2^ = 0.127). Furthermore, Sidak’s *post hoc* test revealed that PD significantly differed among the measurement times [all *p* < 0.0005, baseline, mean = 259.6 mm, SE = 6.4, 95% CI = (246.3, 272.9); Immediately, mean = 183.3 mm, SE = 6.1, 95% CI = (170.6, 196.0); 2 min, mean = 201.8 mm, SE = 7.5, 95% CI = (186.2, 217.5); 4 min, mean = 213.7 mm, SE = 7.9, 95% CI = (197.3, 230.2)] (Table 2 and Figure 3).

**TABLE 2:** Finger position judgment task (proprioceptive drift data).

**Measurement time**	**Synchronous**	**Asynchronous**
Baseline	261.2 ± 35.6	258.1 ± 29.9
Immediately after stimulation	176.3 ± 29.1	190.2 ± 39.9
2 min after stimulation	191.7 ± 44.2	212.0 ± 38.6
4 min after stimulation	204.8 ± 47.0	222.7 ± 37.2

*Distance from the mirror to the estimated left index finger position (mean ± SD) in mm.*

### Correlation

**FIGURE 4:**
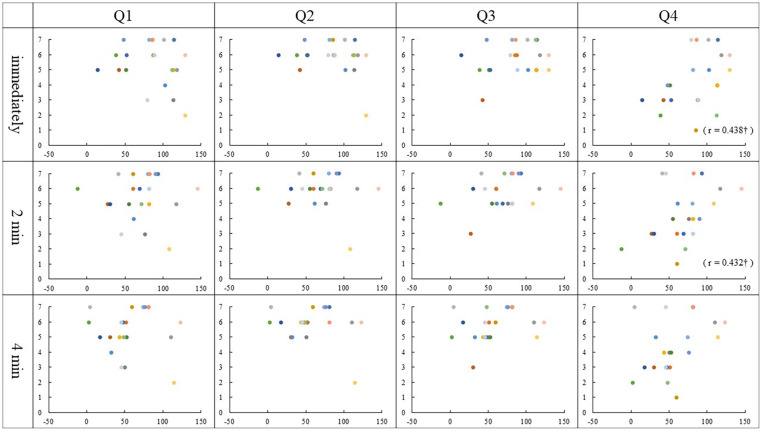
Relationship between PD values and Questionnaire in the synchronous condition: The vertical and horizontal lines indicate the rating scale and the PD value, respectively. The PD values were calculated by subtracting each position after stimulation from the baseline (mm). †*p* = 0.05.

**FIGURE 5:**
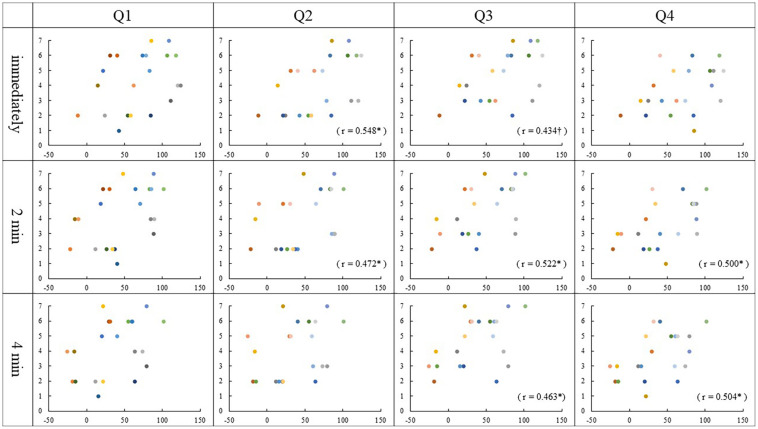
Relationship between PD values and Questionnaire in the asynchronous condition: The vertical and horizontal lines indicate the rating scale and the PD value, respectively. The PD values were calculated by subtracting each position after stimulation from the baseline (mm). ^∗^*p* < 0.05; †*p* = 0.05.

Spearman rank correlation analysis identified that the Q2 ratings correlated with the PD values immediately and 2 min after stimulation in the asynchronous condition (Immediately, *r* = 0.548, *p* = 0.01; 2 min, *r* = 0.472, *p* = 0.03). There was a correlation between the Q3 ratings and the PD values 2 min and 4 min after stimulation, though immediately after stimulation was marginally significant in the asynchronous condition (immediately, *r* = 0.434, *p* = 0.05; 2 min, *r* = 0.522, *p* = 0.02; 4 min, *r* = 0.463, *p* = 0.04). Interestingly, we found that Q4 ratings correlated with the PD values 2 min and 4 min after stimulation in the asynchronous condition (2 min, *r* = 0.500, *p* = 0.02; 4 min, *r* = 0.504, *p* = 0.02). Also, in synchronous condition, the correlations between the Q4 ratings and the PD values immediately and 2 min after stimulation was close to significance (immediately, *r* = 0.438, *p* = 0.05; 2 min, *r* = 0.432, *p* = 0.05). No other correlation was found in either condition (*p* ≥ 0.06) (Figures 4, 5).

## Discussion

In this study, we investigated for the first time whether passive tactile stimulation could induce a body ownership illusion in the mirror image and whether this illusion was persistent. Qualitative evaluation using a questionnaire showed that the degree of body ownership was significantly higher in the synchronous condition than in the asynchronous condition (Q1–3). However, quantitative evaluation by PD measurement revealed that body ownership was elicited and lasted for at least 4 min in both the synchronous and asynchronous conditions. Interestingly, there were correlations between the PD values and some questionnaire items in the asynchronous condition, and then there was no correlation but only one marginally significant one in the synchronous condition. Our study is similar to other studies in that synchronized visual and tactile stimulation was provided and the illusion that body ownership of the participants’ actual hand transfers to the object was induced. In the present study, however, illusory feelings of body ownership were transferred to the mirror image of the right hand rather than the rubber or virtual hand, and tactile stimulation was performed on both hands instead of one. These elements differed from those of previous RHI studies. We, therefore, interpreted that our illusion is different from RHI, which has new insights into multisensory integration.

### Induction of Body Ownership Illusion

The qualitative evaluation revealed that the subjective degree of body ownership was higher in the synchronous condition than in the asynchronous condition for Q1–3. In the synchronous condition, unlike the asynchronous condition, the index finger in the mirror image and the index finger behind the mirror were stimulated not only on the same part but also at the same time. The visual information from seeing the hand in the mirror and the tactile information from the left index finger were integrated owing to their temporal and locational congruence, inducing a “mirror hand illusion” analogous to RHI (Botvinick and Cohen, 1998; Folegatti et al., 2009; Schütz-Bosbach et al., 2009; Lopez et al., 2010; Zopf et al., 2011; Bertamini and O’Sullivan, 2014; Abdulkarim and Ehrsson, 2016). This integration might have strengthened the degree of the qualitative judgment of body ownership, resulting in higher ratings for Q1–3 in the synchronous condition than in the asynchronous condition. In the asynchronous condition, even though the timing of the tactile stimulation applied to the hand in the mirror and the real left hand did not match, the result of Q3 was rated as 5 (somewhat agree), which was higher than we had expected. This question requires judging the rating based on the appearance of the mirror image of the hand, but not on tactile and/or proprioceptive sensation. In the RHI study, participants can recognize that the rubber hand is different from their hand, but in our study, we adopted the mirrored image of the right hand, which extremely resembled the left hand. Previous RHI studies showed that the properties of the rubber hand (such as shape, texture, color, and orientation) affected body ownership (Kilteni et al., 2015; Lira et al., 2017). We, therefore, considered that the participants easily had the illusion that the mirror image was their left hand. Since the rating of Q3 in the synchronous condition was higher than that in the asynchronous one, it is plausible that in addition to the property of the mirror image, matching tactile and visual stimulation timing might have contributed to strengthening the illusory recognition.

In contrast, there was no difference in the results of Q4 between the synchronous and the asynchronous conditions, and the median value of Q4 was 4 or less (“neither” to “disagree”). In the RHI’s previous studies, Q4 was regarded as a control question (Makin et al., 2008; Lopez et al., 2010; Zopf et al., 2011; Abdulkarim and Ehrsson, 2016; O’Dowd and Newell, 2020) and the rating of Q4 tended to be lower than those of Q1–3 in those studies, which was similar to our results. It is speculated that the reason for the low score was due to the content of Q4. We considered Q4 asking about PD-related phenomena and was regarded as an illusion question, to be precise, the question asks about “the experience of the illusory movement,” but not “occurrence of PD.” Actually, throughout the experiment, participants were asked to keep their left hand in the same position and were not allowed to move it. Previous neuroimaging studies of RHI showed that the brain regions related to proprioception were activated when information from the visual cortex and primary somatosensory cortex was integrated into the parietal lobe (Ehrsson et al., 2004; Makin et al., 2007; Tsakiris et al., 2007, 2008), although no activation was found the primary motor area. The results of these activation studies support the idea that the awareness of the hand position changed could emerge without the illusory hand movement. Furthermore, this result was not affected by the tactile stimulation conditions. As in the previous RHI studies (Lopez et al., 2010; Zopf et al., 2011; Abdulkarim and Ehrsson, 2016; O’Dowd and Newell, 2020), Q4 might be a control question even in the study of mirror hand illusion. If the question in Q4 was changed from “I felt as if my hand were drifting toward the mirrored hand” to “I felt as if my hand were near the mirrored hand,” we might have had a different result.

Conversely, the quantitative evaluation showed that PD occurred immediately after stimulation in both conditions. This result differs from our hypothesis based on previous RHI studies. The common feature of both conditions was that the mirrored index finger and the left index finger behind the mirror were stimulated repeatedly at regular intervals, even though the timing of the brush stimulation did not match in the asynchronous condition. In the asynchronous condition of previous RHI studies, the timing of the tactile stimulation to the rubber hand and the participants’ limb was either not constant (Tsakiris and Haggard, 2005; Tsakiris et al., 2007; Schütz-Bosbach et al., 2009; Holle et al., 2011; Riemer et al., 2013), or constant with a stimulation frequency of approximately 0.5 Hz (Zopf et al., 2010; Shimada et al., 2014), slower than our stimulation. In addition, all participants in those studies were stimulated on a unilateral hand only. Those two points differ from the asynchronous condition in our experiment; hence, we interpreted that the asynchronous condition could have the same effect as the synchronous condition when high frequency constant tactile stimulation is repeatedly administered to both fingers.

Our results differed from those reported by Medina et al. (2015). They examined the mirror illusion tapping both fingers simultaneously (synchronous tapping condition), tapping the left and right fingers alternately to the 170-Hz rhythm of a metronome (asynchronous tapping condition), and watching the reflected hand in the mirror (no movement condition). In their study, the largest illusory displacement was found in the synchronous tapping condition, with the smallest illusory displacement occurring in the asynchronous tapping condition. Therefore, the temporal coincidence of the repetitive finger movement appears to play an important role in the induction of body ownership illusion in the mirror image whereas, when using passive tactile stimulation, stimulating the same spot on the participant’s left and right fingers (as performed in our study) is important to induce the mirror illusion.

### Persistence of Body Ownership Illusion

The time course of PD (Figure 3) revealed that in both conditions, the degree of PD was the highest immediately after stimulation, was retained for 4 min and decreased over time. These results indicate that body ownership for the mirrored hand could not only emerge immediately after stimulation but also lasted 4 min, irrespective of the stimulation condition. The qualitative evaluation results showed that the mirror hand illusion had proprioceptive, visual, and tactile aspects. This suggested that since the multimodal illusion of the mirror image of the hand was firmly constructed, the illusion measured by PD might continue for at least 4 min. The decrease in PD over time may be affected by performing this measurement without the hand in the mirror. However, because mirror therapy (Ramachandran et al., 1995) is always performed with a mirror image of the ipsilesional hand, it is presumed that body ownership may be less likely to decline when the hand is visible in front of the mirror.

### Relationship Between the Qualitative and Quantitative Evaluation of Body Ownership Illusion

The relationship between the questionnaire and the PD value showed correlations between some question items and the PD values showed. Interestingly, most of the correlations were found in the asynchronous condition. Here, we describe Q1–3, which showed a difference in ratings between the conditions, and then Q4, which does not show a difference. So far, even in the RHI studies, only one study has discussed the correlation between the subjective judgment by questionnaire and PD in both the synchronous and the asynchronous conditions though results of three question items were combined into one for analysis.

In the synchronous condition, no correlation was found between the results of Q1–3 and any PD value. The results of Q1–3 ratings scored 5 or higher in most participants, regardless of PD value and the variability was small as shown in Figure 4. This means that the synchronous condition may have strongly affected the subjective judgment of the illusion, which may be the reason why the correlation did not occur.

The results of Q1–3 in the asynchronous condition, however, varied among the participants, and those of Q2 and Q3 showed a correlation with PD at two measurement times (Figure 5). It is considered that the asynchronous tactile stimulation revealed the sensitivity of subjective judgment of the illusion in each participant, and as a result, some correlations were found. Bertamini and O’Sullivan (2014) examined the effect of hand appearance on RHI using a fake hand that resembles a real one and a mechanical hand made of metal wires. They demonstrated that the realistic hand was more effective in inducing illusion than the mechanical hand, though the illusion was induced in both hands. In addition, there was a correlation between PD and questionnaire only in the synchronous condition with the realistic hand. This result suggests that using the mirrored hand image may be one factor that caused the correlation between subjective and objective judgment in our asynchronous condition.

On the other hand, the result of Q1 did not correlate with any PD values. Q1 asks, “It seemed as if I were feeling the touch of the paintbrush in the location where I saw the mirrored hand touched.” In this question, participants required comprehensive judgment based on the position of the mirrored hand and that of one’s left hand as well as the timing of tactile stimulation for those hands. As a result, the variability of the subjective judgment became large among participants; then it is presumed that Q1 did not correlate with PD values.

Regarding Q4, some correlations were found with some PD values in the asynchronous condition, then a marginally significant correlation was found with the PD value immediately after tactile stimulation in the synchronous condition. As an individual judgment, the illusion that their left hand had moved toward the mirror image of the right may reflect the result of the estimation based on the PD regardless of stimulation conditions.

### Body Ownership Mechanism

The models of Tsakiris’ top-down process (2010) and Samad et al.’s bottom-up process (2015) are widely known as models of body ownership and body ownership illusion, respectively. In Tsakiris’ model (2010), taking the induction of ownership in a rubber hand as an example, three comparisons (between the body model and the visual form of the rubber hand, between the body state and the posture of the rubber hand, and between the felt touch and the visual information from touching the rubber hand) were made. If participants stated that there was no difference between the two elements in all comparisons, it was considered that a feeling of ownership had been induced in the rubber hand (Tsakiris, 2010).

On the other hand, Samad used Bayesian causal inference to propose that when participants inferred that both temporal (temporal coincidence of tactile stimulation to the rubber hand and participants’ hand) and spatial information (locational coincidence between the rubber hand and the invisible participants’ hand) were related to a common cause, both sources of information were integrated. This induced ownership of the rubber hand. In Samad’s model, even if one of the factors related to the mirror illusion is weak, the other factors including the properties of the rubber hand such as texture, shape, and orientation compensate for the weakness and provide information on the common cause.

In this study, PD was induced to the same extent in both the synchronous and asynchronous conditions. In the asynchronous condition, the visual and tactile information did not match, but the appearance of the mirrored hand and its orientation closely resembled the hand behind the mirror. In addition, the stimulation intervals were consistent with those of the synchronous condition. Therefore, we concluded that these factors might have contributed to the illusion of PD, based on Samad’s model. The results of the questionnaire were, however, rated lower for illusion induction in the asynchronous condition, which was difficult to explain using Samad’s model. Therefore, the explanation in Tsakiris’ model may be more suitable.

These interpretations suggest that quantitative evaluation using PD and qualitative evaluation using the questionnaire may each evaluate different aspects of body ownership.

### Limitations

The findings of this study have to be seen in the light of some limitations.

First, since the present study targeted healthy young participants, the effect of aging on our experiment is not clear (Marotta et al., 2018; Riemer et al., 2019). In addition, we did not control the intellectual function of the participants.

Next, since we adopted a bilateral stimulation approach, it is unclear how tactile stimulation of the right hand alone or no tactile stimulation of both hands affects the PD and subjective judgment. Furthermore, the quantitative evaluation was performed until 4 min after stimulation. It is, therefore, unknown if the illusion can last longer than 4 min. Participants were asked to maintain their eyes closed between PD measurements. We do not know whether continuous looking at the mirror with and without the image of the right-hand affects the degree of PD and its persistence.

Finally, it was not possible to rule out that the participants’ suggestibility (Lush, 2020) might affect their results because no appropriate control question was set for our questionnaire in advance. Also, we did no control whether each item of our questionnaire could properly evaluate the state of the illusion in the mirror image.

## Conclusion

In the present study, the results of PD, which can quantitatively evaluate body ownership, revealed that the same degree of body ownership was induced in both of the synchronous and asynchronous conditions and that it lasted for at least 4 min. Additionally, the body ownership ratings by the questionnaire showed higher in the synchronous condition than in the asynchronous condition, except for one item. We suggest that the difference between qualitative and quantitative evaluations may involve different mechanisms.

In the future, in addition to examining points mentioned in Limitations, introducing neuroimaging research will provide clues for understanding the effect of the tactile stimulation on illusory body ownership and the mechanism of multisensory integration.

## Data Availability Statement

The original contributions presented in the study are included in the article, further inquiries can be directed to the corresponding author/s.

## Ethics Statement

All procedures were conducted in accordance with the Ethical Guidelines for Medical and Health Research Involving Human Subjects and the 1964 Helsinki declaration and its later amendments (seventh revision, 2013) or comparable ethical standards. Written informed consent was obtained from all participants included in this study. The study was approved by the Ethics Committee of the Sapporo Medical University (30-2-35).

## Author Contributions

AI, HS, and HO contributed to conception and design of the study. AI organized the database and performed the statistical analysis. All authors contributed to manuscript revision, read, and approved the submitted version.

## Conflict of Interest

The authors declare that the research was conducted in the absence of any commercial or financial relationships that could be construed as a potential conflict of interest.
